# Detection and Molecular Characterization of *Listeria monocytogenes* and *Listeria* spp. Isolates Recovered From Cattle Farms in Mpumalanga Province, South Africa

**DOI:** 10.1155/vmi/4708466

**Published:** 2025-12-06

**Authors:** Khomotso Confidence Moabelo, Nomakorinte Gcebe, James Gana, Yusuf Bitrus Ngoshe, Rebone Moerane, Abiodun Adewale Adesiyun

**Affiliations:** ^1^ Department of Production Animal Studies, Faculty of Veterinary Science, University of Pretoria, Private Bag X04 Onderstepoort, Pretoria, 0110, South Africa, up.ac.za; ^2^ Bacteriology Department, Onderstepoort Veterinary Research, Agricultural Research Council, Pretoria, South Africa, arc.agric.za; ^3^ Department of Agricultural Education, Federal University of Education, P.M.B. 39, Kontagora, Niger State, Nigeria; ^4^ School of Veterinary Medicine, Faculty of Medical Sciences, University of the West Indies, St. Augustine, Trinidad and Tobago, uwi.edu

**Keywords:** cattle farms, *Listeria*, Mpumalanga, serotypes, South Africa, virulence genes

## Abstract

This study determined the prevalence, characteristics and factors associated with isolating *L. monocytogenes* and other *Listeria* species (*Listeria* spp.) from samples collected from cattle farms in Mpumalanga province, South Africa. A total of 475 samples comprising fresh faeces, pooled environmental faeces, silage, feeds and water were collected from 25 farms (feedlot, cow–calf operations and communal) in three districts (Bronkhorstspruit, Emalahleni and Middleburg). Standard bacteriological and molecular assays were used to isolate, identify and characterize *Listeria* isolates. The prevalence of *L. monocytogenes* and other *Listeria* spp. in farm samples was 2.5% (12/475) and 9.2% (44/475) (*p* < 0.05), respectively. The highest prevalence of isolation of *L. monocytogenes* and *Listeria* spp. was 5.9% (5/85) and 16.7% (5/30) in faeces and silage, respectively. Farm size was the only factor significantly (*p* < 0.05) associated with detecting *L. monocytogenes*; the only serotype detected was 1/2a, and all the isolates were positive for virulence genes *hlyA* and *inlJ.* The overall prevalence of *L. monocytogenes* in samples collected from cattle farms (2.5%) across the province, the detection of serotype 1/2a associated with human listeriosis and the positivity of all strains for one or more virulent genes all pose significant public health and food safety risks from the sources assessed. There is a need to implement measures to reduce or eliminate carriage or contamination by *L. monocytogenes* on cattle farms to avoid the entry of the pathogen into the human food chain in South Africa.

## 1. Introduction

*Listeria monocytogenes* is a Gram‐positive, zoonotic bacterial pathogen of humans and animals that can survive in different environments [1]. *Listeria* spp. are ubiquitous and can be detected in several environments, including soil, water, animal feed, and fresh and frozen meat [2]. *Listeria monocytogenes* is a causative agent of foodborne listeriosis [3], which occurs as both invasive and noninvasive disease [4]. Listeriosis rarely occurs in humans; however, the disease is associated with severe clinical manifestations, such as abortion, preterm birth or stillbirth in pregnant women [5], meningitis or encephalitis, and death [6]. In animals, listeriosis has been reported to cause encephalitis, abortion, mastitis, repeat breeding and endometriosis [7, 8].

The genus *Listeria* currently includes 21 recognized species, comprising *Listeria monocytogenes*, *Listeria seeligeri*, *Listeria ivanovii*, *Listeria welshimeri*, *Listeria innocua and Listeria grayi*, among others [9]. However, only two of these species, *L. monocytogenes* and *L. ivanovii,* are considered pathogens [10, 11]. *L. monocytogenes* is considered one of the important human foodborne pathogens worldwide [12], and *L. ivanovii* is recognized as a mammal pathogen, particularly in ruminants [13].

*Listeria* spp*.* are saprophytic bacteria primarily found in farm environments and are therefore considered part of the typical farm environment [14]. On farms, *L. monocytogenes* has been described as more prevalent in the spring and winter compared to the summer and autumn seasons. On farms, *L. monocytogenes* has been described as more prevalent in the spring and winter than in the summer and autumn [15]. *L. monocytogenes* can infect several types of livestock, such as cattle, sheep, goats and pigs [16]. In cattle, the pathogen can cause ‘circling disease’, encephalitis, meningitis, septicaemia and mastitis [17]. In cattle or other ruminant livestock, *L. monocytogenes* is found in faeces and feed, and the pathogen’s spread on cattle or other ruminant farms is in the form of biofilms [18]. *Listeria monocytogenes* can be excreted in healthy and clinically infected cattle faeces, contaminating farm environments, beef hides and beef carcasses [19]. Listeriosis in cattle is important in the veterinary sector because of its negative impact on animal health, causing premature death or failure of reproduction, and is responsible for economic losses [20].

In farming, affected animals may shed the pathogen in their faeces, allowing the bacteria to circulate [21, 22]. The prevalence of *L. monocytogenes* in bovine dairy and beef operations was between 2.7% and 92%, with silage, hay, bedding and water considered major sources and vehicles of *L. monocytogenes* in the agricultural sector [15, 23]. Poorly fermented silage has been known to facilitate the survival and multiplication of *L. monocytogenes*, which are responsible for listeriosis and are also known as silage disease or cycling disease [24].

As part of classifying pathogens, including *L. monocytogenes*, phenotypic and molecular methods such as polymerase chain reaction (PCR) have been applied to group *L. monocytogenes* into serotypes using flagellar (H) and somatic (O) antigens [25] or serogroups or genoserogroups [26]. To date, 13 *L. monocytogenes* serotypes (1/2a, 1/2b, 1/2c, 3a, 3b, 3c, 4a, 4ab, 4b, 4c, 4d, 4e and 7) have been identified, which are further characterized into serogroups such as IIa (1/2a‐3a), IIb (1/2b‐3b‐7), IIc (1/2c‐3c), IVa (4a‐4c) and IVb (4ab‐4b, 4d‐4e) [27]. However, only four (1/2a, 1/2b, 1/2c and 4b) of the 13 serotypes account for approximately 95% of human listeriosis cases [27].

The pathogenicity of *L. monocytogenes* primarily depends on the virulence genes or factors it carries [28, 29]. Virulence genes that belong to *Listeria* pathogenicity island (LIPI) clusters are essential, such as those in LIPI‐1 (*prfA*, *plcA*, *plcB*, *hlyA*, *mpl* and *actA*) [30] and LIPI‐3 (*llsA, llsB, llsD, llsG, llsH, llsP, llsX* and *llsY*), which facilitate the infectious life cycle and survival in the food or host [31]. Surface‐associated internalins of *L. monocytogenes* play a crucial role in mediating bacterial adhesion and invasion of host cells [29]. Surface‐associated internalins, such as *inlA*, *inlB*, *inlC* and *inlJ*, are responsible for encoding different internalin genes that are involved in the invasion of epithelial cells, and they are also believed to play a role in the tissue tropism of *Listeria monocytogenes* [32, 33] and, in the process, facilitate the ability of the pathogen to survive and spread in the host cells [34].

In South Africa, between 2017 and 2018, the world’s largest outbreak of human listeriosis caused by *L. monocytogenes* [35, 36] was associated with the consumption of ‘polony’, a ready‐to‐eat meat product that is inexpensive, easily accessible and well‐liked by people from all socioeconomic backgrounds in South African cities and rural areas. It is primarily made by South African food manufacturers using mechanically recovered meat (chicken, pork or beef) [37, 38]. In that outbreak, 216 deaths and more than a total of 1060 cases were reported for the period 2017–2018 [39] were documented across the country’s nine provinces, of which 5.1% (11/216) and 4.5% (48/1060), respectively, originated from Mpumalanga province [40]. In South Africa, *L. monocytogenes* has been associated with listeriosis in livestock, notably in goats in the Western Cape [41], where an outbreak occurred in a flock following the introduction of new animals. In the country, the first report of ovine listeriosis was documented in goats in the Western Cape [41]. Subsequently, an ovine listeriosis outbreak was associated with poor flock management practices [42]. Most recently, Gana et al. [43] reported the prevalence of *L. monocytogenes* in cattle farms and abattoirs in Gauteng province. To date, there is a dearth of data on the status of *L. monocytogenes* and other *Listeria* spp. on cattle farms in Mpumalanga province, South Africa.

Therefore, the objectives of this study were to determine the prevalence of *L. monocytogenes* and other *Listeria* spp. in samples collected from cattle farms in Mpumalanga province, South Africa; identify the variables associated with the detection of *Listeria*; and use PCR to determine the occurrence and distribution of the serotypes and virulence genes in *L. monocytogenes* according to the variables investigated.

## 2. Materials and Methods

### 2.1. Study Design

This cross‐sectional study determined the prevalence and molecular characteristics of *L. monocytogenes* and other *Listeria* spp. recovered from cattle farms in Mpumalanga province, South Africa.

### 2.2. Cattle Farms

Information on the cattle farms operating in Mpumalanga province was obtained from the Veterinary Services and Animal Health Assistance in the Gauteng Department of Agriculture and Rural Development (GDARD). Based on the information received and the relative number of each of the three types of farms in the province, it was decided to randomly collect samples from 10, 10 and 5 communal, cow–calf and feedlot operations, respectively. Within each farm, animals were randomly selected for sampling to minimize selection bias and ensure that the collected samples represented the herd. The study was conducted on 25 cattle farms across Mpumalanga province between April 2021 and May 2021.

#### 2.2.1. Description of the Farms

##### 2.2.1.1. Communal Farms

In South Africa, these farms represent small‐scale, subsistence‐oriented production systems in communal areas. In these systems, cattle farming is the major enterprise, accounting for about 75% of production. Herd sizes are generally small, averaging 19 cattle. Farmers mainly keep cattle for meat and cash income (47% of use), with management practices characterized by continuous grazing on rangeland or mountainsides. Most farmers (93.5%) rely on natural veld as the primary feed source, whereas 82% provide periodic supplementation, typically during periods of nutritional stress.

##### 2.2.1.2. Cow–Calf Operations

Cow–calf operations in South Africa form the basis of beef production, with breeding herds managed to produce weaned calves, typically at 5–7 months, for sale to feedlots or backgrounding enterprises. These systems range from smallholder to commercial farms and utilize breeds such as Bonsmara, which are adapted to local conditions. Production is generally extensive or semi‐intensive, relying on grazing with seasonal feed supplementation to address nutritional deficits, especially during dry periods.

##### 2.2.1.3. Feedlot Operations

Feedlots are highly structured, confining cattle and providing high‐energy, grain‐based diets to promote rapid weight gain, contributing significantly to the formal beef supply chains. The average number of cattle per feedlot varies widely, with large commercial operations holding up to 25,000 to 100,000 head of cattle at a time. Cattle sources for feedlots in South Africa primarily include weaners purchased from commercial and emerging farmers. The weaners are usually between 200 and 300 kg when entering feedlots for finishing.

### 2.3. Determination of Sample Size to Be Collected on Cattle Farms

The sample size for the study was estimated using the following formula [44]:

(1) no=1.962×Pexp×1−Pexp d2,

where *P*_exp_ is the expected prevalence and *d* is the desired precision; *P*_exp_ value of 50% and a *d* value of 4.5%; *n*_*o*_ = 3.84 × 0.5 × 0.5/0.0020 = 0.96/0.002 = 480; and *n*_*o*_ = 480 samples.

For the study, 475 samples were collected (5 silage samples were unavailable at five cow–calf operation farms) from 25 cattle farms, consisting of 10 communal farms, 10 cow–calf operations and 5 feedlots across the province. The sample types included faecal samples from individual animals, pooled faecal samples from areas where animals congregate, water from drinking troughs, feeds in feeding troughs, silage and effluents (Table 1). The numbers presented in Table 1 represent the total number of samples collected across all farms within each category. For example, 20 individual faecal samples were collected per farm in five feedlot operations. This resulted in 100 individual faecal samples. The same approach was applied to the other farm categories, where the number of samples collected per farm was multiplied by the number of farms sampled, yielding the overall totals, as shown in Table 1.

**Table 1:** Types and number of samples collected from feedlots, cow–calf operations and communal farms in Mpumalanga Province, South Africa.

Sample type	Number of samples collected at each category of farm:			
	Feedlot operation^†^	Cow–calf operation^‡^	Communal farms^⁑^	Total no. of samples
Faeces (individual animals)	100∗	80	50	230
Pooled faecal samples	25	30	30	85
Water samples from troughs	15	20	10	45
Feeds in feeding troughs	15	20	20	55
Silage	20	10	0	30
Effluents	10	10	10	30
Total no. of samples	185	170	120	475

^†^Samples were collected from 5 feedlot operations.

^‡^Ten cow–calf farms were sampled.

^⁑^Ten communal farms were sampled.

∗Calculated by multiplying the number of samples collected per farm by the number of farms in each category.

### 2.4. Sources, Types of Samples Collected and Transportation to the Laboratory

At selected cattle farms consisting of feedlots, cow–calf operations and communal farms in Mpumalanga, South Africa, the following samples were collected as described:

• *Faeces (rectal faecal grabs or freshly voided faeces from individual cattle): A long-arm glove was used to collect samples of individually selected cattle from the rectum.* In feedlot operations, 20 samples were collected per farm, resulting in 100 samples (20 × 5). In cow–calf operations, eight samples were collected per farm, resulting in 80 samples (8 × 10). In communal farms, five samples were collected per farm, giving 50 samples (5 × 10). Faecal samples were collected via a rectal swab with a rectal glove, placed in a tightly closed plastic cup and put in a sterile plastic bag for transportation to the laboratory.
• *Pooled faecal samples are voided faeces collected from areas where cattle frequently congregate, such as feeding or resting areas:* In feedlots, five samples were collected per farm (25 in total). In cow–calf operations and communal farms, three pooled samples were collected per farm, giving 30 and 30 samples, respectively. Pooled faecal samples were collected using sterile wooden spatulas. Each spatula was carefully inserted into the middle of the faecal pat without contacting the underlying soil to avoid contamination. Different sterile spatulas and collection containers were used for each pooled sample to prevent cross‐sample contamination.
• *Water samples from drinking troughs:* Three samples were collected per farm in feedlot operations (15 in total), two samples were collected per farm in cow–calf operations (20 in total), and one sample was collected per farm in communal farms (10 in total). Water was collected from the trough using a 500‐mL sterile bottle. The bottles were tightly closed and placed in sterile bags.
• *Feeds in feeding troughs:* Three samples were collected per farm in each category, resulting in 15 samples from feedlots (3 × 5), 20 samples from cow–calf operations (2 × 10) and 20 samples from communal farms (2 × 10). Grains and grass were collected from the troughs and placed into tightly closed cups; different types of feed, grain and grass were collected from the farms.
• *Silage:* Four samples were collected per feedlot (20 in total), and one sample was collected per cow–calf farm (10 in total). No silage samples were collected from communal farms. Silage was collected based on availability on the farm and placed into tightly closed sterile cups.

*Effluents and stagnant water (water that is still, often collecting in puddles, ponds or ditches is accessible to cattle on the farms):* Two samples were collected per farm, resulting in 10 samples from feedlots, 10 from cow–calf operations and 10 from communal farms. Pooled water samples from ponds, stagnant water on the farm and effluents on the farm were collected using 500‐mL sterile bottles. The bottles were tightly closed and taken to the laboratory for processing.

The farm samples collected were transported, ice‐cooled within 12 h of collection, to the ARC‐Onderstepoort Veterinary Institution Feed and Food Laboratory and processed within 48 h.

### 2.5. Distribution of Samples Collected From Sample Farms

The distribution of the sample types collected from the three types of cattle farms is shown in Table 2. Of the 475 samples collected, the number varied considerably among the three variables investigated. Across the three districts, the lowest number of samples tested was in Kriel, 11.2% (53/475), while the highest was in Emalahleni, 46.9% (223/475). For the types of farms, the sample size ranged from 25.3% (120/475) in communal farms to 40% (190/475) in feedlots. Regarding the types of samples collected, the least number of samples originated from effluent and silage, 6.3% (30/475), and the highest number, 230 (48.4%) of 475 samples, was from individual faecal samples.

**Table 2:** Distribution of the number of samples collected from Mpumalanga according to the district and the types of farms and samples collected, 2019–2020.

Variable	Level	No. of samples
District	Delmas	199
	Emalahleni	223
	Kriel	53

Type of farm	Communal	120
	Cow–calf operation	165
	Feedlot	190

Type of sample	Effluent	30
	Individual faeces	230
	Pooled faecal samples	55
	Feeds	85
	Silage	30
	Water	45

### 2.6. Isolation and Identification of *L. monocytogenes* and *Listeria* spp.

All samples were analysed using qualitative methods for *L. monocytogenes*, and *Listeria* spp. were tested using the *Listeria* Precis method as described by Matle et al. [45] with minor modifications.

#### 2.6.1. Enrichment of Samples

##### 2.6.1.1. Faecal Samples

Sterile spoons were used to scoop faecal samples from the cups into sterile petri dishes, weighing 10 g of the faecal samples. The samples were transferred aseptically into stomacher bags containing 90 mL of ONE Broth‐*Listeria* (ThermoFisher, South Africa). The samples were homogenized (Stomacher Lab Blender 400, Seward Ltd., West Sussex, UK) at normal speed for 2 min, followed by 48‐h aerobic incubation at 35°C.

##### 2.6.1.2. Feeds

Samples were aseptically removed from the cup using forceps, and 10 g of the feed samples (grass and grain) was weighed using a weighing balance and transferred aseptically into a stomacher bag containing 90 mL of ONE Broth‐*Listeria* (ThermoFisher Scientific, South Africa). This was followed by homogenization and aerobic incubation at 35°C for 48 h.

##### 2.6.1.3. Drinking Water and Effluent Samples

The water centrifugation method was used to isolate *Listeria* spp. from water and effluent samples. For each sample, 100 mL was aliquoted into four 25 mL amounts in centrifuge bottles and then spun down at 13,000 × g for five minutes. The pellets were pooled from the four bottles and inoculated into 9 mL of ONE Broth‐*Listeria* (ThermoFisher Scientific, South Africa) for enrichment, followed by aerobic incubation at 35°C for 48 h. The enriched broth was used to inoculate Brilliance‐*Listeria* agar (BLA) (ThermoFisher Scientific, SA) plates to isolate *Listeria* spp.

#### 2.6.2. Isolation of *Listeria* spp. on *Listeria* Selective Agar

A loopful of enriched broth culture growth in ONE Broth‐*Listeria* (ThermoFisher, South Africa) was inoculated onto BLA plates and streaked for isolation. The inoculated plates were incubated aerobically at 35°C for 48 h. *Listeria* spp. and *L. monocytogenes* were phenotypically confirmed based on characteristic colony morphology on BLA. *Listeria* spp. appeared as blue colonies without a halo, while *L. monocytogenes* appeared as blue colonies with a white/cream halo [46]. Single colonies of suspected *Listeria* spp. and *L. monocytogenes* were subcultured on BLA for further purification. From the initial pool of isolates recovered from BLA plates, 56 were confirmed to be *Listeria* by PCR, which comprised 44 *Listeria* spp., and 12 to be *L. monocytogenes*. The 12 isolates of *L. monocytogenes* were further characterized regarding their serogroups and carriage of virulence genes.

### 2.7. Molecular Identification and Characterization of *Listeria* spp. and *Listeria monocytogenes*

#### 2.7.1. Screening of Suspect Isolates of *Listeria* spp. by Conventional PCR

All enriched broth samples were screened by conventional PCR for *Listeria* spp., i.e., *Listeria* genus. Screening by PCR was performed using an mPCR assay that targets the *prs gene* as previously described by Doumith et al. [47]. The primers used in the current study are shown in Table 3. The PCR products were subjected to electrophoresis on a 3% agarose gel for 3 h at 120 v. *L. monocytogenes* ATCC 19111 was used as a positive control, and water was used as a negative control. The same cPCR assay method was used to characterize *L. monocytogenes* regarding their serotype and virulence profiles.

**Table 3:** Primers used for mPCR serotyping in this study [47].

Assay	Primer	Primer sequence (5′⟶3′)	Product sizes (bp)
mPCR1	*ORF2110*	***ORF2110*-F:** AGTGGACAATTGATTGGTGAA ***ORF2110*-R**: CATCCATCCCTTACTTTGGAC	597
	*ORF2819*	***ORF2819*-F:** AGCAAAATGCCAAAACTCGT ***ORF2819*-R:** CATCACTAAAGCCTCCCATTG	471
	*Imo*1118	***lmo1118*-F:** AGGGGTCTTAAATCCTGGAA ***Imo1118*-R:** CGGCTTGTTCGGCATACTTA	370
	*Imo*0737	***lmo0737*-F:** AGGGCTTCAAGGACTTACCC ***lmo0737*-R:** ACGATTTCTGCTTGCCATTC	691
	*Prs*	***prs*- F:** GCTGAAGAGATTGCGAAAGAAG ***prs*-R:** CAAAGAAACCTTGGATTTGCGG	906

#### 2.7.2. DNA Extraction From Enriched Broth Cultures and Isolates of *Listeria* spp.

DNA was extracted using the boiling‐centrifugation method described by Soumet et al. [48]. Aliquots (2 mL) of enrichment broth were spun at 13,000 × g for 5 min in a centrifuge (Eppendorf, South Africa). The pellets were suspended in 200 μL of sterile bi‐distilled water, heated to 95°C in a dry block for 10 min, cooled at room temperature for 5 min and centrifuged at 13,000 × g for 5 min. The supernatant was pipetted into sterile tubes, and the pellet was discarded. The DNA in the supernatant was then used for further characterization using PCR.

For the isolates of *Listeria* spp., the DNA used for serotyping and virulence gene PCR was extracted from pure cultures as previously described by Matle et al. [45]. Before DNA extraction, the preserved *L. monocytogenes* isolates were revived by inoculation into BHI broth and overnight incubation at 35°C. Loopful from incubated BHI broth was used to inoculate BLA plates, followed by incubation at 35°C for 48 h. Thereafter, 200 μL of sterile distilled water was aliquoted into 2‐mL tubes, and each was inoculated with a loopful of bacterial culture harvested from BLA plates. The bacterial suspension was then vortexed for 10 s, heated at 95°C for 10 min, cooled at room temperature and centrifuged at 13,000 × g for 5 min. The supernatants were transferred into sterile Eppendorf tubes, and debris was discarded. The crude supernatants were stored at −20°C and used as DNA templates in the PCR assays.

#### 2.7.3. Multiplex PCR Assay Used to Classify *L. monocytogenes* Strains Into Serotypes

Multiplex PCR that targets the five fragments of *L. monocytogenes*, namely, imo1118, imo0737, orf2110, orf2819 and prs (specific for *Listeria* spp.), was used to determine the serotypes *L. monocytogenes* as previously described by Doumith et al. [47]. Table 3 indicates the primers and PCR conditions used. *L. monocytogenes* ATCC 19111 was used as a positive control. The products were electrophoresed on a 3% agarose gel, and a gel documentation system (Vacutec, SA) was used to capture the bands.

#### 2.7.4. Detection of Virulence Genes in *L. monocytogenes* Isolates

The presence of selected virulence genes in the isolates of *L. monocytogenes* was determined, as Rawool et al. [49] described. Multiplex PCR was used to detect eight virulence‐associated genes of *L. monocytogenes,* namely, *plcA*, *hlyA*, *actA, inIB*, *iap, inlA*, *inlC* and *inlJ* in two reactions. Reaction 1 (mPCR 1) contained 5 primers (*plcA*, *hlyA*, *actA, inIB* and iap), while Reaction 2 (mPCR 2) consisted of three primer sets (*inlA*, *inlC* and *inlJ*) (Table 4) for the virulence genes. The DNA template preparation from the pathogenic strain of *L. monocytogenes*, PCR assay and agarose gel electrophoresis of the PCR products was performed using the procedure described by Rawool et al. [49].

**Table 4:** Primers used for mPCR virulence profiling in this study [49].

Assay	Primer	Primer sequence (5′⟶3′)	Product sizes (bp)
mPCR2	*InlB*	***inlB*-F:** GATATTGTGCCACTTTCAGGTT ***inlB*-R:** CCTCTTTCAGTGGTTGGGTT	376
	*PlcA*	***plcA*-F:** CTGCTTGAGCGTTCATGTCTCATCCC ***plcA*-R:** ATGGGTTTCACTCTCCTTCTAC	1484
	*HlyA*	***hly*-F:** GTTAATGAACCTACAAGACCTTCC ***hly*-R:** ACCGTTCTCCACCATTCCCA	456
	*ActA*	***actA*-F:** TCGCCGCGGAAATTAAAAAAAGA ***actA*-R:** ACGAAGGAACCGGGCTGCTAG	839
	*Iap*	***iap*-F:** ACAAGCTGCACCTGTTGCAG ***iap*-R:** TGACAGCGTGTGTAGTAGCA	131
	*InlA*	***InlA*-F:** ACGAGTAACGGGACAAATGC ***InlA*-R:** CCCGACAGTGGTGCTAGATT	800
	*InlC*	***inlC*-F:** AATTCCCACAGGACACAACC ***inlC*-R:** CGGGAATGCAATTTTTCACTA	517
	*InlJ*	***inlJ*-F:** TGTAACCCCGCTTACACACAGTT ***inlJ*-R:** AGCGGCTTGGCAGTCTAATA	238

#### 2.7.5. Data Analysis

Laboratory data on the prevalence of *Listeria monocytogenes* and other *Listeria* spp., serogroups and virulence genes from the survey were entered into Microsoft Excel 2016. The data were analysed using Epi Info software (Version 7.0), and the association of variables was determined using Fisher’s exact and chi‐square tests. The level of significance was set at an alpha value of 0.05.

Epi Info was also used to generate percentages for categorical data on the prevalence of *Listeria* spp. in the districts, farm types and sample types; Epi Info also determined the frequency of genoserogroups and virulence genes.

## 3. Results

The overall prevalence of *Listeria* spp. from the farms was 11.8% (56/475, 95% CI: 9.2–15.0). The prevalence of *Listeria monocytogenes* in the 475 samples collected from farms across the three districts was 2.5% (12/475), significantly lower than that of other *Listeria* spp. (non‐*L. monocytogenes*) at 9.3% (44/475) (*p* < 0.001).

The prevalence of *L. monocytogenes* by the district location of the farms, type of farms and sample types is shown in Table 5. The differences in the prevalence of *L. monocytogenes* varied significantly (*p* < 0.05): feedlots, 6.3% (12/190), had a significantly higher prevalence compared to cow–calf farms, 0.0% (0/165). The differences in the prevalence of *L. monocytogenes* by district (*p* = 0.117) and sample type (*p* = 0.141) were not statistically significant. The highest prevalence of *L. monocytogenes* among districts was detected in the Emalahleni at 4.04% (9/223), and among sample types, pooled faecal samples had the highest prevalence at 5.88% (5/85).

**Table 5:** Prevalence of *L. monocytogenes* and *Listeria* spp. in the samples collected from farms and univariate analysis of associated factors.

Variable	Level	No. tested	No. (%) positive for:				
			*L. monocytogenes*	*No. with confirmed serotype 1/2a*	*p* *-*value	*Listeria* spp.	*p* *-*value
District	Delmas	199	3 (1.5)	3	0.117	14 (7.0)	0.236
	Emalahleni	223	9 (4.0)	9		26 (11.7)	
	Kriel	53	0 (0.0)	0		4 (7.6)	

Type of farms	Communal	120	0 (0.0)	0	< 0.05	13 (10.8)	0.487
	Cow–calf operation	165	0 (0.0)	0		17 (10.4)	
	Feedlot	190	12 (6.3)	12		14 (7.3)	

Type of samples	Effluent	30	0 (0.0)	0	0.141	1 (3.3)	0.121
	Individual faeces	230	7 (3.0)	7		19 (8.3)	
	Pooled environmental faeces	55	0 (0.0)	0		6 (10.9)	
	Feeds	85	5 (5.9)	5		12 (14.1)	
	Silage	30	0 (0.0)	0		5 (16.7)	
	Water	45	0 (0.0)	0		1 (2.2)	

For the prevalence of *Listeria* spp., the differences were not statistically significant (*p* > 0.05) for the three variables investigated. The range of prevalence by district was from 7.0% (14/199) in Delmas to 11.7% (26/223) in Emalahleni district (*p* = 0.236). By farm type, prevalence ranged from 7.3% (14/190) in feedlots to 10.8% (13/120) in communal farms (*p* = 0.487). By sample type, prevalence ranged from 2.2% (1/45) in drinking water to 16.7% (5/30) in silage (*p* = 0.121). These results indicate that, although some categories had higher or lower prevalence numerically, the differences were not statistically significant.

The farm prevalence of *L. monocytogenes* and *Listeria* spp. was 12% (3/25) and 44% (11/25), respectively. The difference was statistically significant (*p* = 0.018), indicating that *Listeria* spp. (non‐*L. monocytogenes*) were significantly more prevalent at the farm than *L. monocytogenes*.

All *L. monocytogenes* isolates from the farm samples belonged to serotype 1/2a (100%, 12/12; 2.5%, 12/475 samples). Serotype 1/2a was detected only in feedlot farms, where the prevalence was significantly higher than cow–calf and communal farms (*p* < 0.05). Within feedlots, serotype 1/2a was identified in 58.3% (7/12) of individual faecal samples and 41.7% (5/12) of pooled faecal samples, but this difference was not statistically significant (*p* = 0.568). By district, the distribution was 25% (3/12) in Delmas, 75% (9/12) in Emalahleni and 0% (0/12) in Kriel. Although Emalahleni had the highest numerical prevalence, the differences across districts were not statistically significant (*p* = 0.670).

Regardless of the source of the 12 isolates of *L. monocytogenes*, overall, the frequency of virulence genes was as follows: *hlyA*, 100% (12/12, 95%CI: 73.5–100.0); *inlJ*, 100% (12/12, 95% CI: 73.7–100.0); *inlB,* 41.7% (5/12, 95% CI: 73.5–100.0); *inlC*, 41.7% (5/12: 95% CI: 73.5–100.0); and i*nlA*, 25% (3/12, 95% CI: 5.5–57.2). All 12 isolates of *L. monocytogenes* were negative for virulence genes *actA*, *plcA* and *iap* (Table 6). The detection frequency of virulence genes ranged from 25.0% (3/12) for *inlA* to 100% (12/12) for *hlyA and inlJ*. The predominant multiple virulence genes detected were *hlyA-lniJ (*58.33%, 7/12) and *hlyA-InIA-lnlC-lnlJ* (25%, 3/12).

**Table 6:** Frequency of individual virulence genes in *L. monocytogenes* isolates (*n* = 12).

Virulence gene	No. positive (*n* = 12)	Frequency (%)	95% CI (%)
hlyA	12	100	73.5–100.0
inlB	5	41.7	15.2–72.3
plcA	0	0	0
actA	0	0	0
Iap	0	0	0
inlC,	5	41.7	15.2–72.3
inlJ	12	100	73.5–100.0
inlA	3	25	5.5–57.2

A total of 12 *L. monocytogenes* isolates were obtained from feedlot operations. All isolates carried *hlyA* (100%) and *inlJ* (100%), while *inlB* (41.7%), *inlC* (41.7%) and *inlA* (25%) were less frequently detected. None of the isolates carried *actA*, *plcA* or *iap*. No virulence genes were detected in isolates from cow–calf operations, communal farms or effluents, feeds, silage and water samples (Table 7).

**Table 7:** Frequency of selected virulence genes in *L. monocytogenes* according to the districts and sample types.

Variable	No. tested	*hlyA*	*inlB*	*inlA*	*inlC*	*inlJ*
District						
Delmas	3	3 (100)	0 (0)	0 (0)	0 (0)	3 (100)
Emalahleni	9	9 (100)	5 (55.6)	3 (33.3)	5 (55.6)	9 (100)
Kriel	0	0 (0)	0 (0)	0 (0)	0 (0)	0 (0)
Sample type						
Individual faeces	7	7 (100)	5 (71.4)	3 (42.9)	5 (71.4)	7 (100)
Pooled environmental faeces	5	5 (100)	0 (0)	0 (0)	0 (0)	5 (100)

The only detected serotype from the cattle farms was 1/2a. According to the serotypes, the predominant virulence genes were *hlyA* (100%) and *inlJ* (100%), while the frequency of the virulence genes in the serotype was 41.7%, 41.7% and 25% for *inlB*, *inlC* and *inlA*, respectively.

## 4. Discussion

Listeriosis is a life‐threatening foodborne disease caused by *L. monocytogenes,* and it mainly affects pregnant women, newborns, immunocompromised individuals and older adults [50, 51]. Several reported listeriosis cases have resulted from consuming contaminated foodstuffs, such as meat, meat products, vegetables and dairy products. It cannot be overemphasized that the *Listeria-*contaminated foods that cause human listeriosis result from the ‘farm to fork’ continuum, mainly linked to meat and meat products [52–54]. Therefore, this study focussed on the approach that addressed the prevalence of *Listeria* in cattle farms (cattle, feeds, silage and farm environments), with the potential for cattle exposed to *L. monocytogenes* to contract listeriosis and in slaughtered infected cattle entering the human food chain. The study provides new and valuable information on the prevalence and molecular characteristics of *Listeria* at the cattle farm level in Mpumalanga province, South Africa.

Our current study found that 2.5% (12/475) of the samples collected from cattle farms is the first report of the detection of *L. monocytogenes* on cattle farms in Mpumalanga province. This prevalence is slightly lower than 3.4% (11/328), which was recently reported for cattle farms in Gauteng province [43]. The differences in the pathogen prevalence between cattle farms in both provinces were not statistically significant (*p* = 0.4896). Variable prevalences of *L. monocytogenes* on cattle farms have been reported by others elsewhere, such as 24.4% in New York [55], 19% in Irish farms [56], 27.9% in Jordan [57] and 42.3% in Spain [58]. Interestingly, in Denmark, Skovgaard and Morgen [59] reported a prevalence of 51% for *L. monocytogenes* and 67.5% for other *Listeria* spp. compared with the 2.5% and 9.3% found in the current study. The low prevalence of *L. monocytogenes* found on cattle farms in Mpumalanga province indicates the risk of cattle listeriosis is low in Mpumalanga province, South Africa. The differences in the cattle farm prevalence of *L. monocytogenes* in our study compared with published reports from other countries may be due, in part, to the season, geographic conditions, sanitation in the production phase, and isolation and detection methods [52].

*Listeria* spp., other than *L. monocytogenes*, were detected at significantly higher prevalence, 9.3% versus 2.5% found in our study. Farm‐based studies have similarly documented a higher prevalence of *Listeria* spp. (*L. innocua* and *L. welshimeri*) (11.3%) than *L. monocytogenes* (3.4%) in studies in Gauteng province, South Africa [43]; 58.9% of *Listeria* spp. *L. monocytogenes* (11%) in cattle farms in Latvia [60]; and 12.85 of *Listeria* spp. than 45 of *L. monocytogenes* in Ethiopia [61]. Although *Listeria* spp. was not specifically speciated in our research, it is known that *L. ivanovii* is an important pathogen of ruminants [62], and *L. innocua* has been documented to cause listeriosis in immunocompromised humans [63, 64]. Therefore, the detection of *Listeria* spp. that are non‐*L. monocytogenes* in the current study may have pathogenic potential.

In our study, of the three variables (district, farm size and sample type), only the farm size had a statistically significant effect on the occurrence of *L. monocytogenes.* It is not surprising because, compared to communal farms and cow–calf farms, feedlots rear a high number of cattle under intensively managed systems and receive most of their cattle from auctions and other farms, thus increasing exposure to pathogens, including *L. monocytogenes.* The size and intensively managed farms have been reported to increase the prevalence of bacterial pathogens (*Brucella* spp., *Escherichia* spp.) by others [65, 66]. In contrast, reports elsewhere failed to detect any association between farm size and the occurrence of *Listeria* [43, 67].

Although in our study, the geographical location of the farms and the sample type did not significantly affect the prevalence of *L. monocytogenes* and other *Listeria* spp., some studies have associated these two variables with their occurrence [60, 68].

In the current study, the farm prevalence for *L. monocytogenes* was 12% (3/25), with the feedlots only yielding positive samples, 60% (3/5), and the sample prevalence was 2.5% (12/475). This is the first report on isolating *L. monocytogenes* in a cattle farm‐based study in Mpumalanga province, South Africa. However, Gana et al. [43] earlier documented *L. monocytogenes* prevalence of 3.6% (3/83), 3.4% (5/147) and 3.1% (3/98) in samples collected from communal, cow–calf and feedlot operations, respectively, in Gauteng province in the country. To date, information on livestock listeriosis in South Africa is limited. Although the prevalence of *L. monocytogenes* (2.5%) in cattle in the current study is low, it is pertinent to mention that an outbreak of listeriosis in sheep and cattle fed unmarketable potatoes was earlier reported in Mpumalanga province [69], where the current study was conducted. Therefore, *Listeria* at the cattle farm level cannot be ignored since cattle can serve as sources of human listeriosis [70]. Earlier, the first report of ruminant listeriosis was documented in goats in the Western Cape [41]. Subsequently, Meredith and Schneider [42] reported an outbreak of ovine listeriosis associated with poor flock management practices.

It is essential that all the silage samples in the current study were negative for *L. monocytogenes,* although 16.7% were positive for *Listeria* spp. Silage has been reported to be the possible source of *Listeria* spp. on cattle farms [71, 72]. Also, poorly fermented silage has been documented to harbour pathogens that cause cattle diseases [73], thus posing a threat to public health [74]. The finding that all the silage samples processed in the current study, all of which originated from only feedlots, were negative for *L. monocytogenes* is indicative that it may not be an important source of pathogens for cattle reared on these farms.

Regarding the other types of samples collected from the cattle farms, only faecal samples (individual animals or pooled) yielded *L. monocytogenes* at a prevalence of 3.8% (12/315) compared with *Listeria* spp. which were isolated from faecal samples (9.8%), effluents (3.3%), feeds (10.9%) and drinking water (2.2%). Animal faeces have been reported to be potential sources of *L. monocytogenes* [75], but the prevalence has varied among cattle farms and countries [67, 72]. The type of cattle farms was documented to affect the prevalence of faecal shedding of *L. monocytogenes* when Mohammed et al. [76] reported a higher prevalence in calf–calf (3.1%) than in feedlots (0.3%), which is the reverse of the findings in the current study, 0.0% and 6.28%, respectively. In Trinidad and Tobago, Adesiyun et al. [77] reported a lower cattle faecal prevalence of *L. monocytogenes* (3.3%) than found in the current study (9.8%), while a comparable prevalence (7.1%) was documented in Jordan [18]. However, a higher prevalence (18.2%) was reported in Slovenia by Bandelj et al. [23], compared to what was found in our study. Depending on the country, different risk factors have been associated with the faecal prevalence of *L. monocytogenes*. While Mohammed et al. [76] did not observe the effect of season, Bandelj et al. [23] reported that the shedding of *L. monocytogenes* in cattle faeces was associated with environmental temperature and meteorological season. There is a possibility of differences in farm management and hygienic practices, and isolation or detection methods affecting the differences in the faecal prevalence of *L. monocytogenes* cannot be ignored. Like the current study, where *L. monocytogenes* was not recovered from the farm effluents despite recovering the pathogen from the faecal samples of cattle (3.8%) on the farms, Adesiyun et al. [77] also did not detect the pathogen from the effluents of farms where 3.3% of the cattle were faecal shedders of *L. monocytogenes* in Trinidad and Tobago. The findings may be attributed to the low frequency of cattle shedders of *L. monocytogenes* in both studies.

In the current study, the only serotype (1/2a) from feedlot samples was 1/2a (2.5%). Gana et al. [43] recently detected that in 11 isolates of *L. monocytogenes* recovered from cattle farms in Gauteng province*,* serogroups 1/2a‐3a (72.7%) and 4b‐4d‐4e (27.3%), a similar finding of serotype 1/2a at a high frequency (72.7%) compared with the 100% detected in our study. Also in agreement with our findings is the report of a survey conducted in the USA by Borucki et al. [71], who reported serotype 1/2a as the predominant serotype from samples collected from cattle in the USA. The predominance of serotype 1/2a in cattle farms indicates that cattle could be potential sources of listeriosis in South Africa. The predominance of 1/2a has been demonstrated to contribute to its ability to form biofilms [78] and its high resistance to sanitizer and bacteriocins [79]. This has been documented as a zoonotic serotype because of its implication in human zoonoses [80].

The current findings provide helpful information about the dominant serotypes in cattle farms in Mpumalanga province, South Africa. However, variable predominant serotypes of *L. monocytogenes* have been reported by others, such as serotypes 1/2a, 1/2b and 4b, documented to make up 78% of the typable isolates of *L. monocytogenes* in Ireland [56], serotypes 1/2a and 4b in Uruguay [81] and serotypes 1/2a (13.2%) and 4b (84.2%) in Spain [82]. The variable frequency of the serotypes of *L. monocytogenes* across cattle farms in various countries may reflect the strains of the pathogens circulating on the farms, management practices such as the use and quality of silage fed to cattle, biosecurity measures for environmental contamination and the adaptability of serotypes to specific farm environments, which ultimately affect the serotypes of a pathogen that spills over into the food chain [79, 83].

Of potential pathological significance is our finding that the isolates of *L. monocytogenes* recovered from cattle farm samples were positive for five (*hlyA, inlJ, inlC, inlB* and *inlA*) of the eight virulence genes assayed for frequencies that ranged from 25% to 100%. It is vital to have detected *hlyA* in 100% of the isolates of *L. monocytogenes* in our study. This is because this virulence gene belongs to the LIPI‐1 gene cluster and other LIPI‐3 genes, which have been reported to play a significant role in the host cell’s infectious cycle [31]. The most commonly reported virulence genes for serotype 1/2a include *prfA, hly, plcA, plcB, mpl, actA, inlA, inlB, inlC, inlJ, iap* and *fbpA* [84, 85]*.* In our study, all isolates carried *hlyA* and *inlJ*, whereas *actA*, *plcA* and iap were absent, and *inlA*, *inlB* and *inlC* were detected at lower frequencies. In an earlier study conducted on cattle farms in Gauteng province, all the isolates were carriers of seven (*hlyA, inlB, plcA, iap, inlA, inlC* and *inlJ*) of the eight genes tested for [43]. It has also been reported that internalins (*inlA, inlC* and *inlJ*) found in our isolates are involved in the pathogenesis of human listeriosis by facilitating host cell surface attachment by the pathogen [86, 87]. In agreement with the findings in the current study, where all (100%) the isolates of *L. monocytogenes* were positive for *hlyA* and *inlJ,* the detection of a similar predominance of only two virulence genes (*inlA* and *inlC*) in cattle farms in Jordan [18] and the presence of internalin genes (*inlA, inlC* and i*nlJ*) in farm isolates in Poland [88]. The potential clinical importance of *L. monocytogenes* strains isolated from cattle faecal samples can, therefore, not be ignored should the contamination of cattle carcasses occur during slaughter, with the potential to enter the human food chain. It is known that several *L. monocytogenes* virulence factors, including listeriolysin O, phospholipases, proteins and several internalins, have been identified and characterized at molecular and cellular levels [89–91]. The ability of *L. monocytogenes* to survive and multiply in its host cells has been reported to be significant for the pathogen’s pathogenicity [91, 92]. Therefore, the detection of *L. monocytogenes* in samples from cattle in the current study possessing virulent genes may have food safety implications. All the strains of *L. monocytogenes* assessed were negative for three virulence genes, *plcA*, *iap* and *actA,* which may have resulted from spontaneous mutations [93].

## 5. Conclusion

The current study demonstrated the presence and distribution of *L. monocytogenes* and *Listeria* spp. in various sample types collected from cattle farms and retail in Mpumalanga province, South Africa. The prevalence (2.5%) of *L. monocytogenes* on cattle farms indicates the risk of human exposure to the pathogen following slaughter and contamination of carcasses of cattle that are carriers of the pathogen and consumption of improperly cooked *L. monocytogenes*‐contaminated beef and beef products. The detection of a zoonotic pathogenic serotype (1/2a) and virulence gene (*hlyA*) of the LIPI‐1 and four internalins (*inlJ, inlC, inlB* and *inlA*) in *L. monocytogenes* isolates poses the risk of listeriosis in cattle and human consumers of contaminated beef and beef products following the slaughter of cattle from these farms.

Future studies should be conducted on cattle farms in the nine provinces of South Africa to fully elucidate the prevalence, characteristics and genomic relatedness of *L. monocytogenes* and all *Listeria* spp. detected using whole genome sequencing.

## Ethics Statement

This study was approved by the University of Pretoria Animal Ethics Committee (AEC), Project number (REC138‐19), on February 24, 2020; the Research Ethics Committee (REC), Project number (REC138‐19), on November 29, 2019; and the Department of Agriculture, Land Reform and Rural Development’s Director of Animal Health (DALRRD) Section 20 approval under Act 35 of 1984.

## Consent

Before sampling commenced, the researchers obtained the consent of the managers or owners of the cattle farms (communal, cow–calf and feedlots).

## Conflicts of Interest

The authors declare no conflicts of interest.

## Funding

The study was funded by the Red Meat Research and Development South Africa (RMRD‐SA) (awarded on January 1, 2019) and the Health and Welfare Sector Education and Training Authority (HWSETA).

## Data Availability

The data supporting this study’s findings are available at upspace@up.ac.za and http://repository.up.ac.za, reference number 4870.
